# Anticoagulation Dilemma in a Patient With Pulmonary Embolism and Massive Haemoptysis

**DOI:** 10.7759/cureus.116889

**Published:** 2026-09-25

**Authors:** Isaac Mugwano, Faith Atim Emilly, Isaac Ssinabulya, David Salama Kaishusha, Joanita Nalyazi

**Affiliations:** 1 Adult Cardiology, Uganda Heart Institute, Kampala, UGA

**Keywords:** body mass index (bmi), massive haemoptysis, obesity, oral anticoagulation, pulmonary embolism (pe)

## Abstract

The development of worsening haemoptysis in a patient with pulmonary embolism presents a significant treatment challenge, especially in a low-resource setting where advanced interventions such as bronchial artery embolization may not be readily available.

We present the case of a 40-year-old morbidly obese woman who was transferred to our facility with recurrent lower-limb deep vein thrombosis and a new diagnosis of bilateral pulmonary embolism. Following initiation of oral anticoagulation with apixaban, she developed worsening haemoptysis that persisted despite dose adjustments. She was subsequently stabilized and discharged on a vitamin K antagonist (warfarin). This case highlights the clinical challenges encountered in managing anticoagulation in the setting of significant bleeding and underscores the importance of individualized anticoagulation therapies.

## Introduction

Venous thromboembolism (VTE), which includes deep vein thrombosis (DVT) and pulmonary embolism, is the third most common acute cardiovascular syndrome globally, following myocardial infarction and stroke worldwide [1].

The development of massive haemoptysis in a patient with pulmonary embolism presents a significant clinical challenge, especially in resource-limited settings where advanced interventions may not be readily available. There are inconsistencies in the definition of massive haemoptysis based on the volume of expectorated blood, with many authors implying a blood loss in the range of 100-1000 mls in twenty-four hours or 150 mls per hour [2-6]. The major pitfall with this definition is the inaccuracies in quantification of blood volumes by patients who are likely to exaggerate or underestimate these quantities.

This observation has given rise to the “magnitude of effect” that defines massive haemoptysis as the expectoration of a life-threatening volume of blood with significant clinical consequences, such as airway obstruction, hypotension, or substantial blood loss. This definition emphasises the severity of massive haemoptysis based on its physiological consequences rather than the absolute volume of blood expectorated. Clinically, massive haemoptysis may necessitate blood transfusion, hospitalisation, endotracheal intubation, or mechanical ventilation due to hypoxemia (PaO_2_ < 60 mmHg). It may also result in aspiration of blood into the contralateral lung, airway obstruction, respiratory failure, and/or even death [7-11].

Our patient was successfully transitioned to and maintained on a vitamin K antagonist (warfarin) with a therapeutic international normalised ratio (INR) at discharge. She remains generally stable on subsequent outpatient follow-ups with continued therapeutic anticoagulation.

Our case highlights the clinical challenge of managing pulmonary embolism with massive haemoptysis, emphasising the need to prioritise bleeding control, reassess thrombotic risk, and individualise anticoagulation, particularly in patients with obesity and limited resources.

## Case presentation

A 40-year-old female patient on antiretroviral therapy since 2000 with good viral suppression, previous deep venous thrombosis of the right leg in 2022 and morbid obesity was transferred from the emergency unit with a computed tomography (CT) scan diagnosis of bilateral pulmonary embolism. At admission, her symptoms included haemoptysis (estimated at 30 ml in 24 hours), chest pain, shortness of breath and worsening general body weakness for one week. She denied any history of fevers, syncope or limb swelling. She denied any history of smoking but regularly drank alcohol. She was a hairdresser and a tailor with long hours of sitting. She had a history of herbal medicine use prior to her current admission. In the emergency unit, she had been initiated on low molecular weight heparin (LMWH), antibiotics and had a chest tube on the left side, which contained 700 ml of straw-coloured pleural effusion. On physical examination, the patient was acutely ill with a body temperature of 37.0 degrees Celsius and morbidly obese with a body mass index (BMI) of 49.1 kg/m^2^. Her radial pulse rate was 98 beats/min, blood pressure was 106/63 mmHg, respiratory rate was 28 breaths/min, and oxygen saturation was 95% when given 15 litres/min of oxygen via a non-rebreather mask.

Her chest examination revealed a dull percussion note in the left infrascapular area with coarse crackles in the right infra-axillary and mammary areas; the chest tube was in situ but with minimal drainage. Her cardiovascular examination noted a loud pulmonary component of the second heart sound and a systolic murmur that was most appreciated at the lower left sternal border.

Initial laboratory workup was notable for thrombocytosis and a mild leucocytosis, while other cell lines were within normal ranges; biochemical analysis revealed mild liver enzyme derangements and a marked systemic inflammatory response, with C-reactive protein and procalcitonin levels reaching 11 and 14 times the upper limit of normal, respectively. The thyroid function results indicated potential subclinical thyroid dysfunction with an elevated thyroid-stimulating hormone (TSH) and a borderline low free triiodothyronine (FT3) (Table 1).

**Table 1:** Complete blood count and chemistry WBC: white blood cell count; Neut#: absolute neutrophil count; Lym#: absolute lymphocyte count; HGB: haemoglobin count; HCT: haematocrit; PLT: platelet count; CRP: C-reative protein; PCT: procalcitonin; TSH: thyroid-stimulating hormone; FT4: free thyroxine; FT3: free triiodothyronine; T. Chol: total cholesterol; LDL: low density lipoprotein; HDL: high density lipoprotein; TG: triglycerides; sCr: serum creatinine; AST: aspartate aminotransferase; ALT: alanine aminotransferase; GGT: gamma-glutamyl transferase; T. Bil: total bilirubin; INR: international normalised ratio; HbA1c: glycated haemoglobin

Parameter	Result	Reference range
WBC	10.71	4.00-10.00 10^9^/L
Neut#	6.17	2.00-7.00 10^9^/L
Lym#	3.5	0.80-4.00 10^9^/L
HGB	12.1	12.0-16.0 g/dL
HCT	34.5	40.0-54.0%
PLT	586	100-300 10^9^/L
CRP	5.72	0.00-0.50 mg/dL
PCT	0.72	0.00-0.05 ng/mL
TSH	8.319	0.350-5.100 µIU/mL
FT4	1.19	0.50-1.40 ng/dL
FT3	1.73	1.80-4.20 pg/mL
T. Chol	113	0.0-160.0 mg/dL
LDL	106	0.0-130.0 mg/dL
HDL	18	40.0-60.0 mg/dL
TG	87	0.0-150.0 mg/dL
sCr	1.12	0.70-1.00 mg/dL
Urea	42.7	0.0-50.0 mg/dL
Sodium	133	130.0-147.0 mmol/L
Potassium	4.9	3.30-5.50 mmol/L
Chloride	101	95.0-112.0 mmol/L
Albumin	3.1	3.90-5.00 g/dL
AST	99	0.0-31.0 U/L
ALT	94.8	0.0-41.0 U/L
GGT	148	0.0-38.0 U/L
T. Bil	0.22	0.30-1.20 mg/dL
INR	1.33	0.76-1.15
HbA1c	6.3	<5.7%
Vitamin B_12_	650	177-664 pmol/L
CD4 count	1219	500-1500 cells/µL

Despite the patient’s history of HIV, her CD4 count was well maintained at 1,219 cells/µL. Extensive infectious work-up for tuberculosis was negative; however, pleural fluid analysis revealed a scanty growth of *Acinetobacter baumannii *and was inconclusive for malignancy.

The ECG showed an S1Q3T3 pattern, with widespread T-wave inversions (Figure 1).

**Figure 1:**
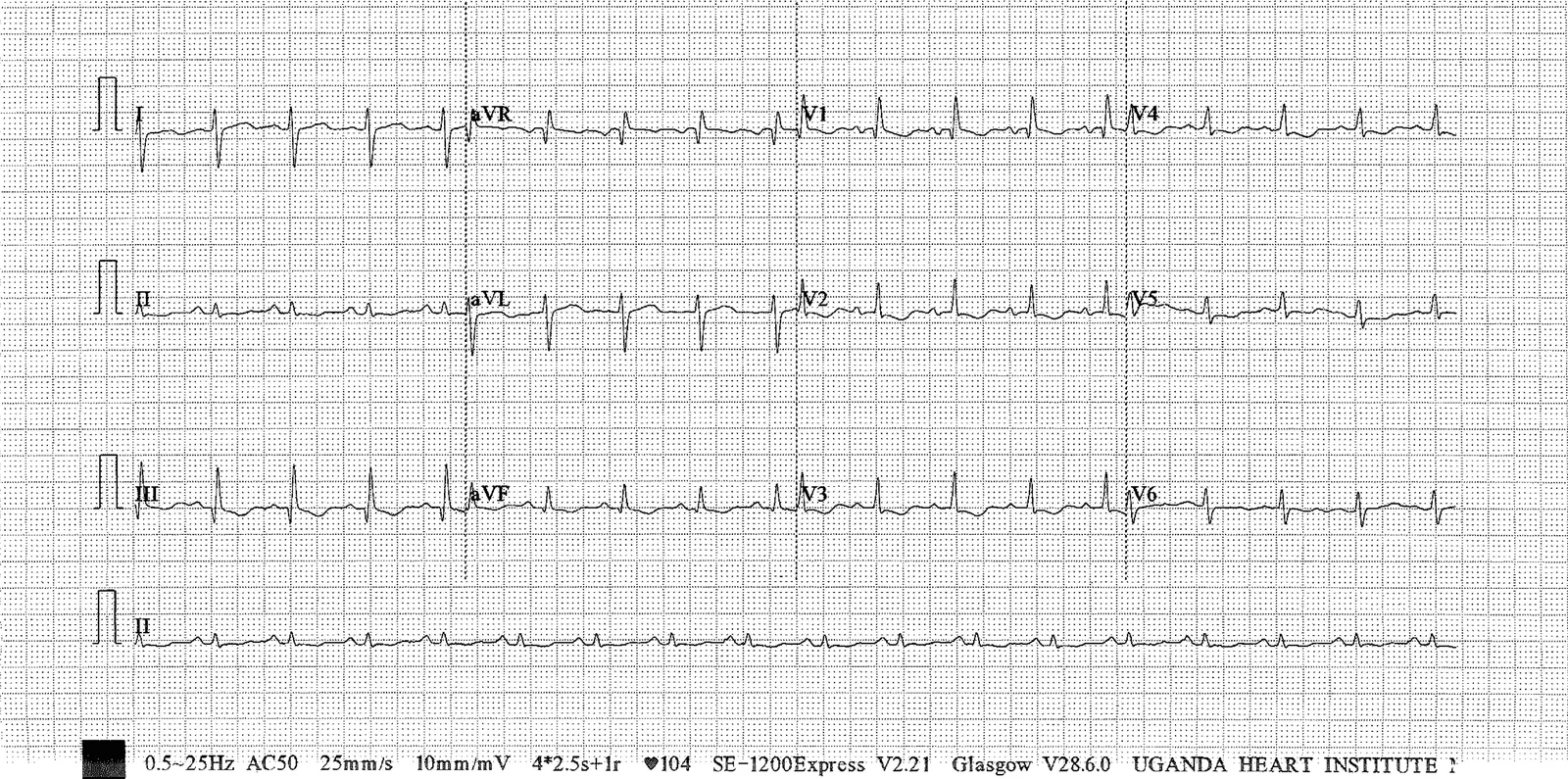
ECG demonstrating sinus tachycardia with features of right heart strain such as right axis deviation, right ventricular hypertrophy, and S1Q3T3 pattern; widespread T-wave inversions are also noted

Echocardiography revealed a dilated right ventricle (5.5 cm) with reduced systolic function (tricuspid annular plane systolic excursion (TAPSE), 1.2 cm) (Figure 2A; Video 1), a D-shaped left ventricle with systolic flattening but preserved systolic function (Video 2), a dilated inferior vena cava with less than 50% collapsibility (Figure 2B), and moderate tricuspid regurgitation with a right ventricular systolic pressure of 69.69 mmHg (Video 3; Figure 2C).

**Figure 2:**
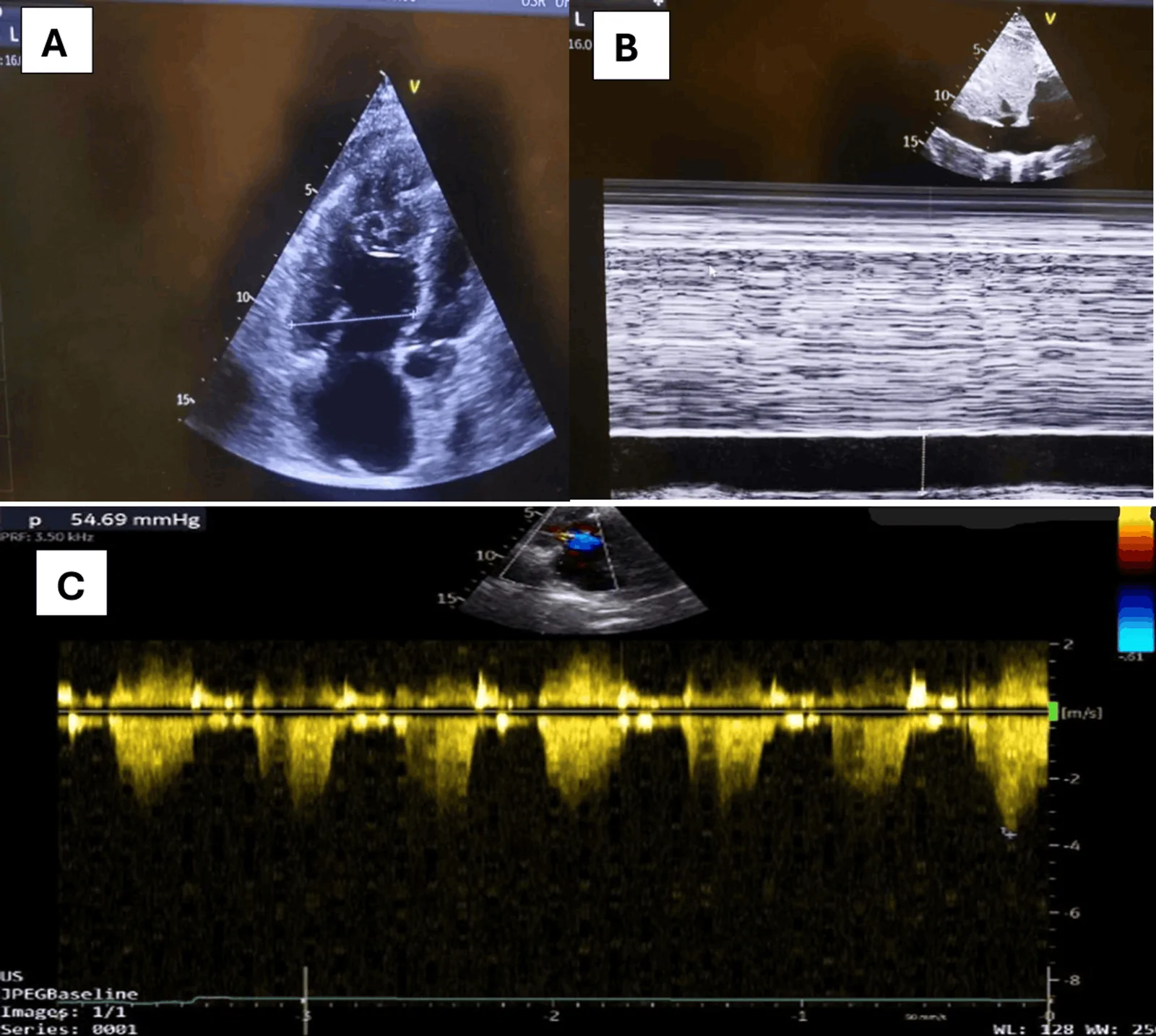
Cardiac echocardiogram (A) Dilated right ventricle and right atrium. (B) Dilated inferior vena cava with less than 50% collapsibility. (C) Tricuspid regurgitation pressure gradient of 54.69 mmHg, estimated right atrial pressure of 15 mmHg, and right ventricular systolic pressure of 69.69 mmHg, indicating severe pulmonary hypertension.

**Video 1:** Transthoracic echocardiographic parasternal long axis view demonstrating right ventricular dilation

**Video 2:** Transthoracic echocardiographic view showing a D shaped left ventricle with systolic flattening of the interventricular septum

**Video 3:** Dilated right ventricle with moderate tricuspid regurgitation

The computed tomography pulmonary angiography (CTPA) revealed extensive bilateral pulmonary emboli with significant thrombus burden noted in the left pulmonary artery (LPA) (Figure 3A), with a dilated main pulmonary artery (Figure 3B) and dilated right ventricle and atrium (Figure 3C), and the lower lobar and segmental branches bilaterally.

**Figure 3:**
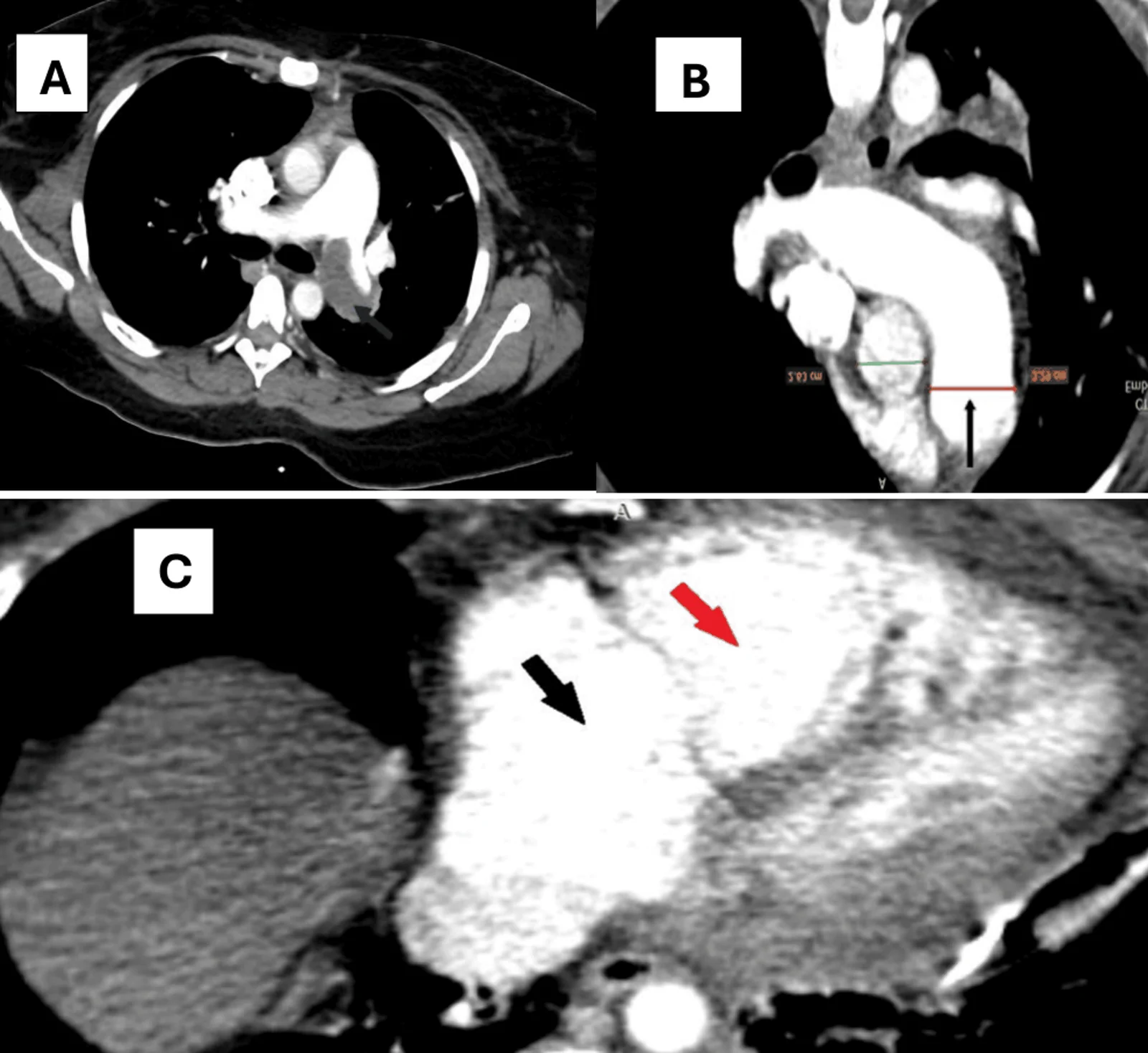
Computed tomography pulmonary angiography (A) Demonstrates the left main pulmonary artery thrombus (dark grey arrow). (B) Shows a dilated main pulmonary artery (black arrow). (C) Shows a dilated right ventricle (red arrow) and a dilated right atrium (black arrow).

The lower limb venous Doppler scan revealed a deep venous thrombosis involving the external iliac vein (Figure 4) and the common femoral veins (Figure 5).

**Figure 4:**
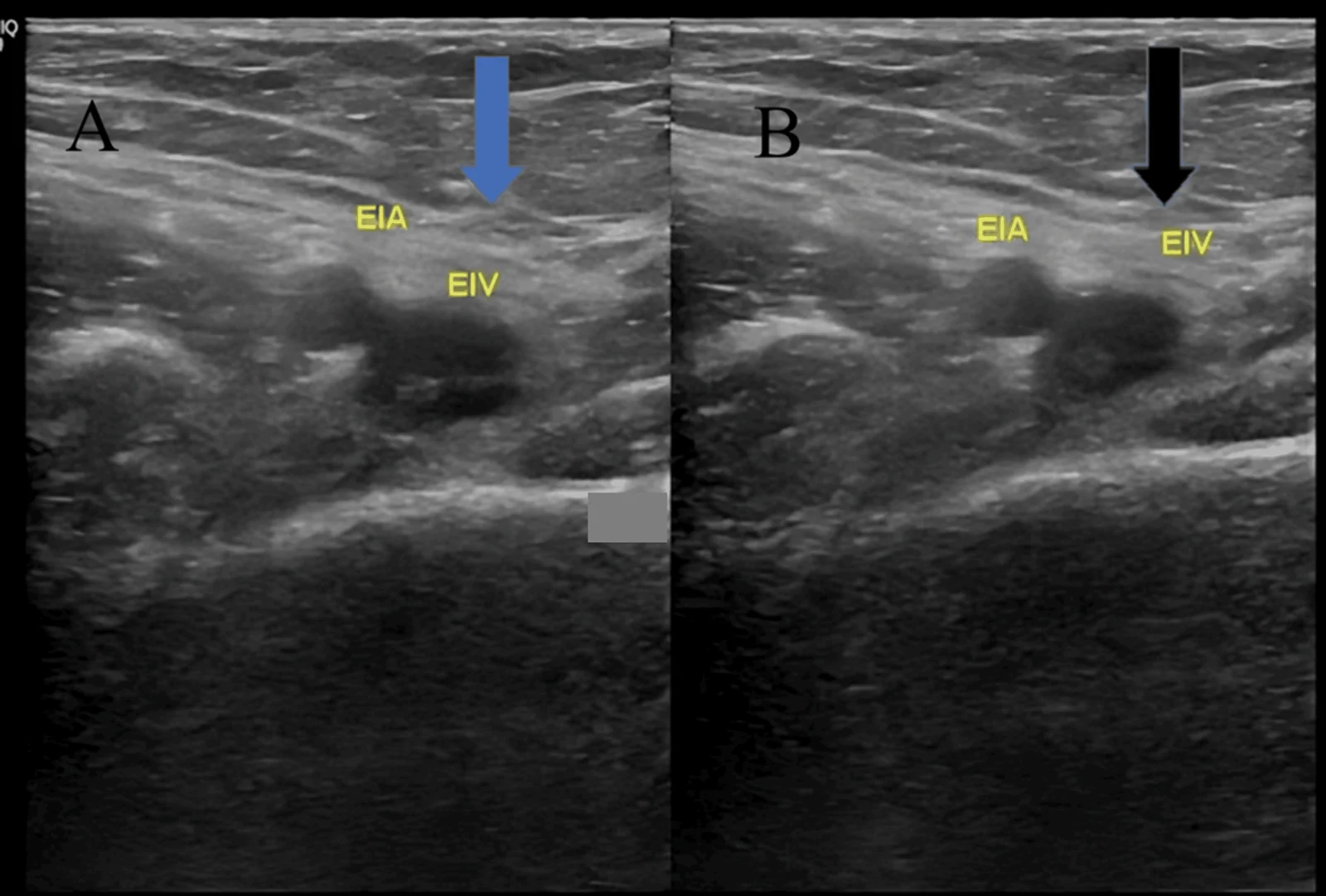
Lower limb venous Doppler scan showing external iliac vein (A) Shows the state before the compression technique (blue arrow). (B) Demonstrates the non-compressibility of the external iliac vein (black arrow). EIA: external iliac artery; EIV: external iliac vein

**Figure 5:**
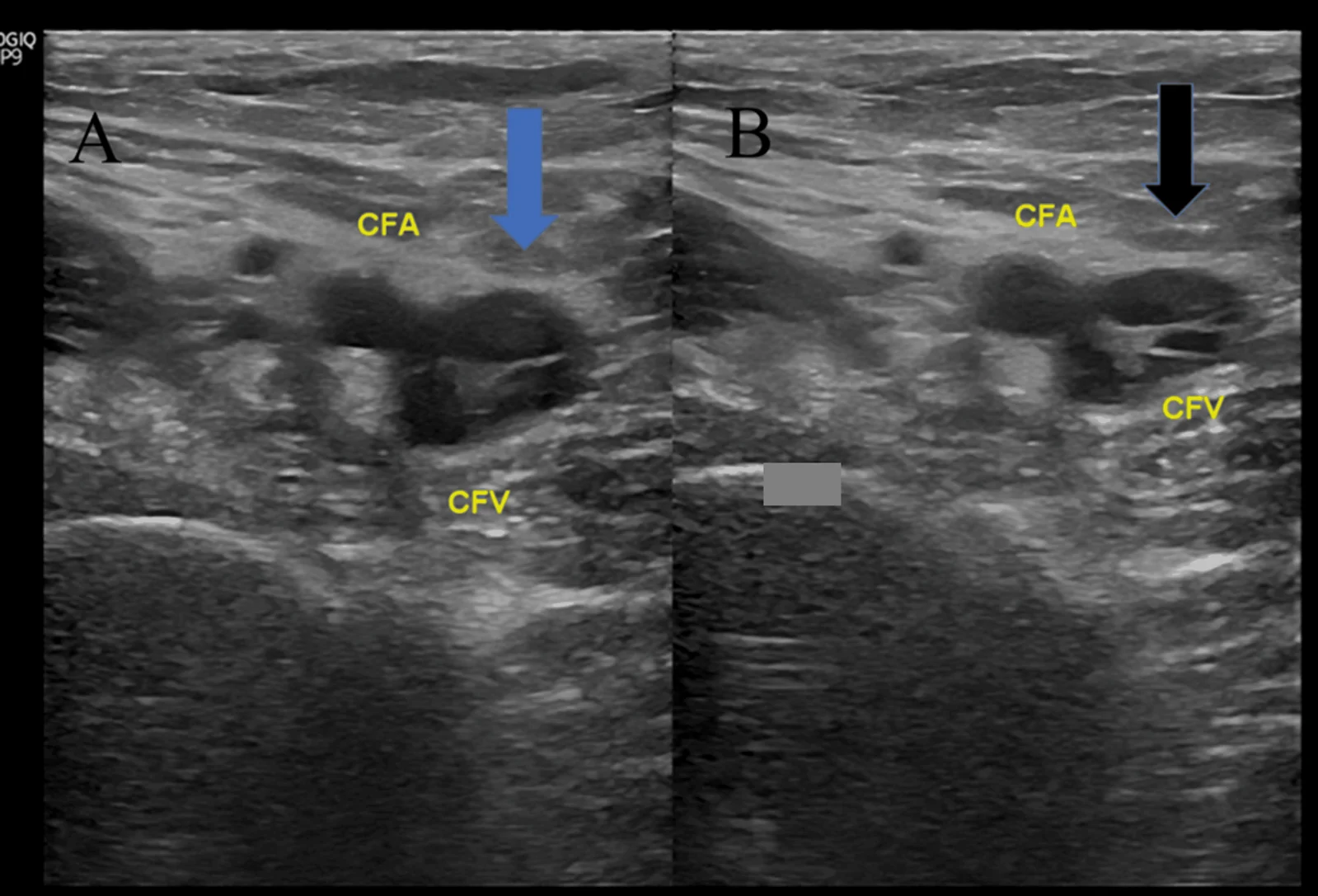
Lower limb venous Doppler scan showing the common femoral vein (A) Shows the common femoral vein before the compression technique (blue arrow). (B) Shows the non-compressibility of the common femoral vein (black arrow). CFA: common femoral artery; CFV: common femoral vein

The chest X-ray revealed moderate pleural effusion on the left side (Figure 6).

**Figure 6:**
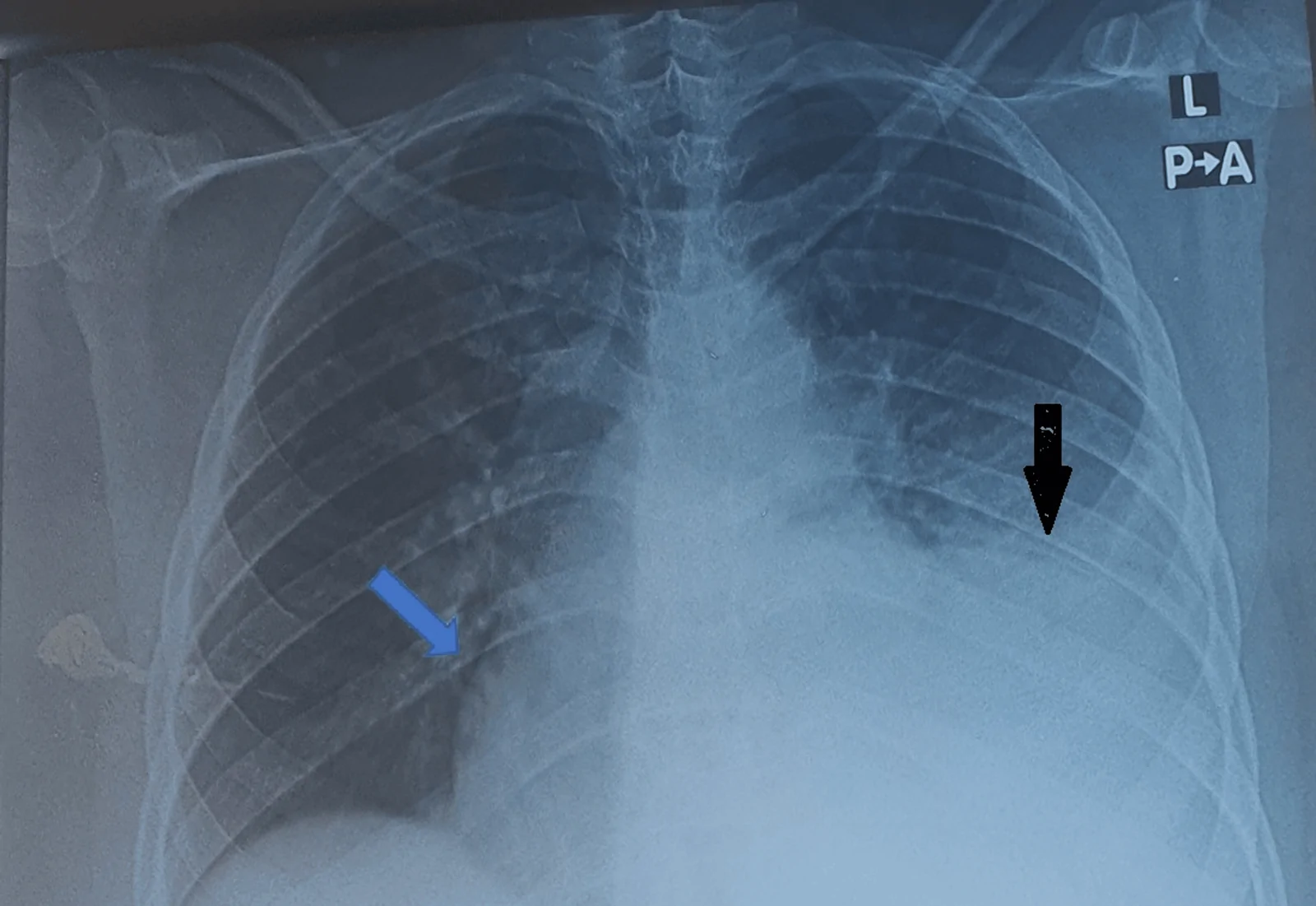
Chest X-ray showing a dilated right atrium (blue arrow) and a moderate left pleural effusion (black arrow)

She was managed with low molecular weight heparin (LMWH), piperacillin/tazobactam (as guided by culture results), oxygen therapy and analgesics. The chest tube was removed on the 3rd day, and the patient switched to apixaban 10 mg twice daily on the 4th day. Her bleeding risk was 1.5 as calculated from the VTE-BLEED score [12], which categorised her as low risk. However, she developed worsening haemoptysis (about 350 ml per hour) and syncope on the 7th day, necessitating a blood transfusion, use of tranexamic acid and high-flow oxygen with a non-rebreather mask. Apixaban was held at this point, and the patient was switched back on LMWH. There were no changes in her neurological status or stool colour to suggest central nervous system or gastrointestinal bleeding, respectively. She improved, and seven days later she was rechallenged with apixaban 5 mg twice daily following three days without haemoptysis episodes, but it was held the following day because she developed a recurrence of haemoptysis. At this point, she was reinitiated on LMWH, pending haematology review. We were not in a position to assay for anti-factor Xa so as to establish the levels of anticoagulation at the time. In consultation with the haematology team, a decision was made to initiate her on warfarin, which she tolerated well, achieving a therapeutic INR of 2.16 in five days, and she was subsequently discharged in stable condition. She has had several outpatient visits subsequently with no major concerns and continues to maintain a stable therapeutic INR.

## Discussion

Background

The major risk factors for venous thromboembolism (VTE), as documented by the European Society of Cardiology (ESC), include hospitalisation for heart failure or atrial fibrillation/flutter within the previous three months, hip or knee replacement surgery, major trauma, myocardial infarction within the previous three months, previous VTE, lower limb fracture and spinal cord injury [13,14]. These factors are categorised as being strong risk factors for thromboembolism. In the case presented above, a history of previous VTE and the coexisting infections, HIV and pneumonia, are classified as moderate risk factors according to the ESC classification. The other factors present in this case, including immobility and obesity, are considered weak risk factors for VTE.

Obesity is associated with persistent, low-grade inflammatory states characterised by increased concentrations of C-reactive protein (CRP) and pro-inflammatory cytokines, including interleukin-6 (IL-6), interleukin-8 (IL-8), and tumour necrosis factor-alpha (TNF-α), which are well recognised drivers of thrombosis. The risk of thrombosis in obesity is further amplified by elevated levels of leptin that is known to trigger platelet aggregation and initiate oxidative stress in the vascular endothelium [15,16]. Although obesity is a well-established risk factor for pulmonary embolism, several observational studies have reported that obese patients with pulmonary embolism have better overall survival than their non-obese counterparts. This unexpected observation, commonly referred to as the "obesity paradox," remains incompletely understood, and the mechanisms underlying this apparent survival benefit continue to be debated in the literature [17,18].

Evaluation and management

In situations of worsening haemoptysis, the first intervention should be resuscitation of the patient, which involves maintaining airway patency, optimising oxygenation and ensuring that the haemodynamics are stable. Once this is achieved, the attending team should attempt to localise the bleeding site as well as establish any possible aetiologies of the bleeding, including interruption of ongoing anticoagulation [3].

Bronchoscopic localisation with either flexible fibreoptic bronchoscopy [19] or rigid bronchoscopy allows for therapeutic interventions including epinephrine instillation, application of thrombin/thrombin-fibrinogen solutions by topical means and endobronchial tamponade therapy [3,20].

In patients who continue to experience persistent haemoptysis despite endobronchial therapies, bronchial arteriography and bronchial arterial embolisation should be considered. There is an indication for surgery in those patients who continue to have persistent bleeding, haemodynamic instability and significant respiratory system dysfunction despite the above interventions [21].

Our patient had registered clinical improvement on LMWH and other supportive treatment, but upon initiation of apixaban, she developed worsening haemoptysis. At the time of decompensation, we wished to perform a CT angiography to identify the location of haemorrhage, but this involved transferring the patient out of the facility, which wasn’t feasible given the patient's clinical status, and we were not in a position to do bedside bronchoscopy [22,23].

The introduction of the novel oral anticoagulants has transformed the paradigm of anticoagulation, with sufficient evidence that they have a faster onset of action, a shorter plasma half-life, minimal interactions with drugs/foods, a predictable dose-response relationship and a lower risk of adverse events. The significant challenge with these agents is the absence of a reversal agent in many facilities, especially in the low- and middle-income countries [24,25].

Evidence from a retrospective registry-based cohort study indicated that, among patients being treated for atrial fibrillation and/or VTE, bleeding events were frequently encountered with rivaroxaban, while apixaban demonstrated the lowest incidence [26]. Despite such a safety profile of the novel oral anticoagulants, we noted that our patient experienced life-threatening bleeding on apixaban, which was not the case when she was switched to warfarin. 

At presentation, our patient was noted to have morbid obesity (BMI, 49.1 kg/m^2^). However, obesity may not necessarily have heightened her risk of anticoagulation-related bleeding, as evidenced by findings from several landmark trials, including ARISTOTLE and ROCKET AF, which did not demonstrate an increased risk of major bleeding among obese patients being treated with direct oral anticoagulants [27,28].

A post hoc analysis of the AMPLIFY trial, which evaluated the efficacy and safety of apixaban in patients with VTE, including those with high body weight or obesity, noted that apixaban was not only safe but was also effective across all weight categories, including patients weighing ≥ 120 kg or with BMI > 40 kg/m^2^ [29].

They also observed similar VTE recurrence rates and lower bleeding risks compared to enoxaparin/warfarin, even though higher body weight correlated with slightly lower (but not clinically significant) drug exposure. These findings support using apixaban in obese patients without requiring dose adjustments based on weight alone [29].

However, a cohort study evaluating the association between BMI and bleeding risk among elderly patients with non-valvular atrial fibrillation receiving dabigatran demonstrated a significant positive linear relationship between BMI and bleeding risk. The study found that each 1 kg/m² increase in BMI was associated with a 12% increase in the risk of bleeding (P = 0.021) [30].

Similar findings were reported in a retrospective study evaluating clinical outcomes of patients with atrial fibrillation and elevated BMI who were on direct oral anticoagulants (DOACs). The study included patients treated with dabigatran, apixaban or rivaroxaban, and classified them into non-obese, class I obesity, and class II or greater obesity groups based on BMI. The study noted that the patients with higher BMI experienced stroke or systemic embolism significantly earlier and developed major bleeding episodes sooner compared with those with lower BMI. The findings, although contrary to those documented from the ARISTOTLE, ROCKET AF, and AMPLIFY trials, may partly explain the occurrence of bleeding events noted in this patient at the time she was switched to apixaban [27-29,31].

The risk of recurrent VTE in obese patients was further evaluated in a post hoc analysis of the EINSTEIN trials [32]. The analysis included patients with severe obesity and demonstrated no significant difference in the risk of recurrent VTE between those treated with rivaroxaban and those receiving warfarin. These findings were consistent across higher body weight categories, including patients weighing 120-140 kg and those weighing ≥140 kg [32].

The pharmacokinetics of DOACs in obese patients were investigated in a prospective observational study that assessed patients across different weight categories, including patients < 50 kg, 50-120 kg, and those above 120 kg. The study proceeded to evaluate plasma concentrations of rivaroxaban, bleeding events, and recurrent VTE during the follow-up period. The authors found that rivaroxaban plasma levels in patients with severe obesity were comparable to those seen in individuals with normal body weight [33].

While there is a weight-based dose adjustment for LMWH, the optimum dosing for novel oral anticoagulants is not well established in obese patients. In a systematic review of 20 studies conducted by Abildgaard et al. in PubMed and Embase, it was noted that rivaroxaban and apixaban can be used in obese patients with VTE or atrial fibrillation, including those patients with BMI > 40 kg/m^2^, at standard fixed doses [34].

The 2016 guideline from the International Society on Thrombosis and Hemostasis emphasised that DOACs could be used as standard therapy for the treatment of VTE, prevention of recurrent VTE, and prevention of stroke in patients with non-valvular atrial fibrillation and a BMI of ≤40 kg/m^2^ or body weight ≤120 kg. However, the same body in 2021 updated its guideline and recommended that direct oral anticoagulants, especially apixaban and rivaroxaban, may be used at standard doses for the treatment and prevention of VTE regardless of a patient’s body weight or BMI, indicating the emergence of additional evidence supporting their efficacy and safety in patients with obesity [35].

Further evidence regarding the choice of anticoagulant therapy in patients with higher BMI has been provided by a multicenter retrospective cohort study conducted by Dobry et al. [36]. The study included 1,736 patients with a mean body weight of 164.4 kg and an average BMI of 54.6 kg/m^2^. The study investigated the safety and effectiveness of direct oral anticoagulants, specifically apixaban and rivaroxaban, as potential alternatives to vitamin K antagonist warfarin for the treatment of atrial fibrillation and/or VTE. Based on their findings, the investigators concluded that apixaban and rivaroxaban were comparable to warfarin in terms of safety and effectiveness, supporting their use as alternative anticoagulant options for patients with atrial fibrillation and/or VTE who have a higher BMI of ≥50 kg/m² and/or body weight ≥150 kg [36].

Similar findings were also reported by O'Kane et al. in their retrospective cohort study, who noted that apixaban and rivaroxaban were safe and effective in the prevention of thromboembolic events and posed no increased bleeding events among patients with BMI ≥50 kg/m² with non-valvular atrial fibrillation [37].

The patient received tranexamic acid, which works by inhibiting plasminogen activation. The drug has limited evidence for use in patients presenting with massive haemoptysis; however, in a retrospective cohort study, Kinoshita et al. evaluated the association between tranexamic acid administration and mortality among patients with haemoptysis requiring emergency admission. Of the 28,539 patients included in the study, 60% received tranexamic acid, while 40% did not [38]. They noted that patients who received tranexamic acid had significantly lower in-hospital mortality and shorter hospital stays compared with those who did not receive tranexamic acid [38].

## Conclusions

The worsening haemoptysis in patients undergoing anticoagulation with novel oral anticoagulants for a life-threatening condition like pulmonary embolism creates a unique clinical dilemma that involves interruption of anticoagulation, urgent resuscitation and the search for a possible aetiology. Whereas the clinical course points to a temporal association between apixaban exposure and worsening haemoptysis, there is not enough evidence to allude to causation.

There is also currently insufficient evidence to determine whether the patient's morbid obesity contributed to the recurrence of venous thromboembolism or the subsequent worsening of haemoptysis while receiving apixaban. Further randomised controlled trials are needed to investigate these potential associations.
